# Enhancing Choices Regarding the Administration of Insulin Among Patients With Diabetes Requiring Insulin Across Countries and Implications for Future Care

**DOI:** 10.3389/fphar.2021.794363

**Published:** 2022-01-14

**Authors:** Ileana Mardare, Stephen M. Campbell, Johanna C. Meyer, Israel Abebrese Sefah, Amos Massele, Brian Godman

**Affiliations:** ^1^ Public Health and Management Department, Faculty of Medicine, “Carol Davila” University of Medicine and Pharmacy Bucharest, Bucharest, Romania; ^2^ Division of Population Health, Health Services Research and Primary Care, University of Manchester, Manchester, United Kingdom; ^3^ Division of Public Health Pharmacy and Management, School of Pharmacy, Sefako Makgatho Health Sciences University, Pretoria, South Africa; ^4^ Pharmacy Practice Department of Pharmacy Practice, School of Pharmacy, University of Health and Allied Sciences, Volta Region, Ghana; ^5^ Pharmacology and Therapeutics Department, Hurbert Kairuki Memorial University, Dar Es Salaam, Tanzania; ^6^ Centre of Medical and Bio-allied Health Sciences Research, Ajman University, Ajman, United Arab Emirates; ^7^ Strathclyde Institute of Pharmacy and Biomedical Sciences, University of Strathclyde, Glasgow, United Kingdom

**Keywords:** Africa, cost-effectiveness, central and eastern European countries, evidence-based decisions, hypoglycaemia, insulin pumps, patient choices, type 1 diabetes

## Abstract

There are a number of ongoing developments to improve the care of patients with diabetes across countries given its growing burden. Recent developments include new oral medicines to reduce cardiovascular events and death. They also include new modes to improve insulin administration to enhance adherence and subsequent patient management thereby reducing hypoglycaemia and improving long-term outcomes. In the case of insulins, this includes long-acting insulin analogues as well as continuous glucose monitoring (CGM) systems and continuous subcutaneous insulin infusion systems, combined with sensor-augmented pump therapy and potentially hybrid closed-loops. The benefits of such systems have been endorsed by endocrine societies and governments in patients with Type 1 diabetes whose HbA1c levels are not currently being optimised. However, there are concerns with the low use of such systems across higher-income countries, exacerbated by their higher costs, despite studies suggesting their cost-effectiveness ratios are within accepted limits. This is inconsistent in higher-income countries when compared with reimbursement and funding decisions for new high-priced medicines for cancer and orphan diseases, with often limited benefits, given the burden of multiple daily insulin injections coupled with the need for constant monitoring. This situation is different among patients and governments in low- and low-middle income countries struggling to fund standard insulins and the routine monitoring of HbA1c levels. The first priority in these countries is to address these priority issues before funding more expensive forms of insulin and associated devices. Greater patient involvement in treatment decisions, transparency in decision making, and evidence-based investment decisions should help to address such concerns in the future.

## Introduction

The global burden of diabetes is substantial and growing (Godman et al., 2021a; Godman et al., 2021b). There was an estimated 463 million people with diabetes mellitus worldwide in 2019 (Chan et al., 2021), with prevalence rates expected to grow to 578 million by 2030 (International Diabetes Federation, 2019), enhanced by rising incidence rates (Liu et al., 2020). This is a concern as diabetes is associated with appreciable morbidity, including health-related quality-of-life (HRQOL), mortality and costs, with the costs of treating patients with diabetes enhanced by the cost of associated complications (Smith-Palmer et al., 2016; Almeida et al., 2018; Bommer et al., 2018; Rwegerera et al., 2018; International Diabetes Federation, 2019; Li et al., 2019; Stedman et al., 2020; Chan et al., 2021). Complications associated with diabetes include non-traumatic lower-extremity amputations, blindness, and cardiovascular disease (CVD), with patients with diabetes also at a greater risk of end-stage renal disease (Einarson et al., 2018; International Diabetes Federation, 2019; Godman et al., 2021a; Chan et al., 2021). Poor management of patients with diabetes can increase their risk of CVD by up to 3-fold, with up to 30% of patients with diabetes dying from CVD (Benjamin et al., 2019; Chan et al., 2021). The combined direct and indirect costs of treating patients with diabetes and their complications globally in 2015 was estimated at US$1.3 trillion, with the combined costs projected to reach US$2.1–US$2.5 trillion by 2030, equating to 2.2% of Gross Domestic Product (GDP) (Bommer et al., 2018; Roze et al., 2021).

Whilst most people with diabetes have Type 2 diabetes (T2DM), an appreciable number of patients have Type 1 diabetes (T1DM) (Godman et al., 2020a; Godman et al., 2021c). In addition, some patients with T2DM, especially in low- and middle-income countries (LMICs), will require insulin to control their diabetes (Baruah et al., 2017; Venkataraman et al., 2020; Godman et al., 2021c). Overall, it was estimated in 2019 that over 1.1 million adolescents worldwide had T1DM, including over 600,000 children under 15 years of age (Patterson et al., 2019), with an average increase in incidence rates of 3–4% per year (Tuomilehto et al., 2020). This is particularly important across countries including Africa, with rising numbers of patients with diabetes including those requiring insulin, as well as among European countries including the United Kingdom, which has one of the largest numbers of new cases of T1DM annually among European countries in children aged 14 years and younger (Godman et al., 2020b; Roze et al., 2021). This is because there are concerns with late diagnosis and treatment of T1DM among children especially in among African countries as well as other LMICs, which results in avoidable high mortality rates from diabetic ketoacidosis, severe hypoglycaemia, infection and eventually microvascular complications (Godman et al., 2020b; Godman et al., 2021b) adding to the global burden of diabetes. Such concerns are enhanced among LMICs where there can be high co-payments for medicines including those to treat patients with diabetes (Haque et al., 2021a; Godman et al., 2021b; Haque et al., 2021b; Godman et al., 2021c) alongside high costs for monitoring HbA1c levels negatively impacting on care (Godman et al., 2020b). As a result, access programmes and support from donor organisations are often needed among LMICs, especially African countries, to enhance the affordability of even basic insulins, as well as monitoring of HbA1c levels, among patients, and their families along with measures to enhance earlier diagnosis (Shannon et al., 2019; Godman et al., 2020b; Godman et al., 2021b; Haque et al., 2021b). This includes donor schemes in Kenya to reduce the price of insulin Mixtard^®^ 1000IU by two-thirds to enhance usage (Shannon et al., 2019; Godman et al., 2021b).

There is improved diagnosis and management of T1DM in higher income countries, with mortality rates from T1DM appreciably decreasing in recent years (Tuomilehto et al., 2020). Similarly, we have seen glucagon-like peptide-1 receptor agonists when added to metformin-based therapy in patients with T2DM appreciable reduce HbA1c levels (Tsapas et al., 2020). There have also been reductions in mortality when patients with T2DM at increased CV risk have been prescribed newer therapies including dapagliflozin, liraglutide, and semaglutide alongside metformin. Newer medicines including liraglutide and semaglutide have also reduced CV deaths in patients with T2DM, with sodium-glucose cotransporter-2 inhibitors shown to reduce hospitalization due to heart failure and end-stage renal disease (Tsapas et al., 2020). However, such medicines are typically unaffordable in a number of LMICs including African countries (Godman et al., 2020a). Key aspects of care in patients with T2DM in these countries are focused on prevention alongside making sure key medicines, including oral metformin, are readily accessible (Godman et al., 2020a). We have seen in South Africa that there is limited funding for dialysis in patients with T2DM in the public system with the authorities preferring to support preventative measures within limited funds (Makhele et al., 2019). We are also aware that children with T1DM are still at higher risk of death compared with the non-diabetic population even in high-income countries, especially those with diabetic nephropathy (Tuomilehto et al., 2020), and diabetes can also have a negative impact on patients’ quality of life (Currie et al., 2006; Almeida et al., 2021; Roze et al., 2021).

Consequently, patients with diabetes, including children, especially if they require insulin, should be increasingly carefully managed and monitored. This should include enhancing potential choices of available insulins, and their administration, to improve patient convenience, and subsequent adherence to prescribed treatments, cognisant of issues of affordability, especially if there are concerns with multiple injections with standard insulins such as NPH insulins as well as cultural issues. Greater choice and involvement of patients in decision making, especially with respect to issues surrounding injection frequency, should help to reduce the extent of hypoglycaemia and associated complications as well as improve long-term outcomes (Inzucchi et al., 2012; Pfützner, 2017; Fifer et al., 2018; Muñoz-Velandia et al., 2019). However, only funded when standard insulins such as NPH insulins, and accompanying monitoring equipment, are readily available and accessible within public healthcare systems without appreciable co-payment issues (Godman et al., 2020b; Haque et al., 2021b). Improved glycaemic control has been shown to reduce the risk of morbidity and mortality in patients with diabetes as well as associated costs (Nathan, 2014; Mortality in Type 1 Diabe, 2016; Chan et al., 2021), with fear of hypoglycaemia negatively impacting on patients’ quality of life (McCoy et al., 2013; Roze et al., 2021).

Long-acting insulin analogues were developed to reduce the risk of hypoglycaemia, especially nocturnal hypoglycaemia, producing greater patient convenience through reducing the number of injections (Pedersen-Bjergaard et al., 2014; Rys et al., 2015; Godman et al., 2021a; Chan et al., 2021; Tricco et al., 2021). Whilst there have been concerns over their additional costs versus standard insulins such as NPH insulins, and whether this represents value (Caires de Souza et al., 2014; Almeida et al., 2018; Ewen et al., 2019; Hemmingsen et al., 2021), recent published studies, including systematic reviews, have shown that their higher acquisition costs can be offset by savings from averted costs associated with hypoglycaemia and other complications (Jendle et al., 2020; Lee et al., 2020; Shafie and Ng, 2020; Tricco et al., 2021) generating medium to long-term savings. As a result, long-acting insulin analogues are now the most widely prescribed insulins among high-income and high middle-income countries, with sales growing in other countries including Central and Eastern European (CEE) countries as well as Bangladesh and India (Silver et al., 2018; Ewen et al., 2019; Godman et al., 2021a; Haque et al., 2021a; Godman et al., 2021c; Godman et al., 2021d). This is welcomed as there have been concerns in CEE countries regarding the availability and funding of biological medicines to treat patients with rheumatoid arthritis, psoriasis, and inflammatory bowel disease (Putrik et al., 2014; Kostic et al., 2017; Baumgart et al., 2019). Among CEE countries, including Bosnia and Herzegovina, the Czech Republic, and Estonia, there has been high use of long-acting insulin analogues versus other forms of insulin, with high expenditure on long-acting insulin analogues also seen in Romania in recent years (Godman et al., 2021d; Tubic et al., 2021), with this trend continuing.

Building on the situation observed in other disease areas, the increasing availability of biosimilars of long-acting insulin analogues should help to reduce their costs and increase their use, (Haque et al., 2021a; Godman et al., 2021e; Godman et al., 2021f). However, this is not always the case with insulin glargine, with the originator company dropping its price to compete, as well as limited price reductions in practice for biosimilar insulin glargine in a number of countries including CEE countries (Godman et al., 2021a; Godman et al., 2021d). In addition, three companies currently dominate the insulin market worldwide in terms of both usage and expenditure, making competition difficult (Beran et al., 2018; Godman et al., 2021a; Godman et al., 2021b). This is starting to change.

The continued higher prices for long-acting insulin analogues, including biosimilars, versus standard insulins such as NPH insulins, has limited their use in practice among a number of countries (Godman et al., 2021b; Haque et al., 2021b). This includes many African and South American countries (Godman et al., 2021a; Godman et al., 2021b). However, increased local production of insulins, building on examples in other LMICs as well as concerns with supplies of medicines during the current COVID-19 pandemic, are potential ways forward to enhance the availability and use of insulins including long-acting insulin analogues as seen in Brazil and Malaysia (Fiotec, 2015; Ogunleye et al., 2020; Godman et al., 2021b; Godman et al., 2021c). In addition, the World Health Organisation (WHO) has recently launched a pre-qualification scheme for insulins to increase competition and help lower prices (WHO, 2019). This is welcomed as prices for long-acting insulins among the public healthcare systems in Africa and South America need to be close to standard insulins such as NPH insulins to enhance future funding and use (Godman et al., 2021a; Godman et al., 2021b).

The WHO, World Bank and the Organisation for Economic Co-operation and Development have advocated five foundational elements critical for delivering quality health services in the goal of achieving universal healthcare for all by 2030. This is underpinned by governments, policymakers, health system leaders, patients and clinicians all working together (WHO, 2018). Key areas include the provision of safe and most effective use of medicines as well as ensuring a high quality healthcare workforce (WHO, 2018). Within this, it is important that healthcare professionals discuss treatment options available to patients with diabetes requiring insulin when reviewing different management approaches accessible and available within their healthcare systems. This includes the different types of insulin and their administration, and the importance of adherence to prescribed treatments. As part of effective management strategies, it is also imperative that healthcare professionals fully engage with children and adolescents, as well as parents and guardians in the case of young children, when discussing available insulins and their associated dosing regimens including skin hygiene. This is especially important among children and adolescents who have a fear of hypoglycaemia, or poor baseline glycaemic control, aiming to improve their care when self-administering insulins. The ultimate goal of treatment being to try and mimic the production of insulin as closely as possible, with the minimum number of injections and monitoring (Shah et al., 2016; Roze et al., 2021), especially considering studies showing high rates of hypoglycaemia in patients with diabetes requiring insulin (Heller et al., 2020; Pinés Corrales et al., 2021). However, any additional activities and costs associated with educational programmes for different insulin regimens must be factored into their overall costs when making funding decisions within public healthcare systems (Christie et al., 2016). This includes the cost for any associated educational and other activities including appropriate tools among patients with diabetes and any caregivers to enhance adherence to prescribed medicines (Christie et al., 2016; Iqbal et al., 2017; Kyokunzire et al., 2018; Liu et al., 2021).

### Developments in Insulin Administration and the Implications

There have been developments in the administration of insulin over the years, attempting to reduce the burden of multiple daily administration of insulin and concomitant constant monitoring (Shah et al., 2016). Advances include the potential for oral insulin (Wong et al., 2018) as well as potentially buccal or transdermal insulin (Shah et al., 2016). In addition, the development of continuous glucose monitoring (CGM) systems to provide information about blood glucose levels and glycaemic trends in real time to patients, given concerns with the levels of unawareness regarding hypoglycaemia among some patients (Smith et al., 2009; Rodbard, 2017; Ajjan et al., 2019). However, there can still be episodes of severe hypoglycaemia (Lin et al., 2019) and concerns with the level of health gain with CGM systems versus the costs involved; though, this is not universal (Health Quality Ontario, 2018; Wan et al., 2018).

Alongside this, the development of continuous subcutaneous insulin infusions (CSII) systems combined with sensor-augmented pump therapy (SAP) and potentially hybrid closed-loops (HCL) systems to respond to changes in HbA1c levels thereby further reducing episodes of hypoglycaemia (Garg et al., 2017; Karges et al., 2017; Steineck et al., 2017; Burckhardt et al., 2018; Gómez et al., 2018; Korkmaz et al., 2018; Stone et al., 2018; Grunberger et al., 2021; Moreno-Ferández et al., 2021; Roze et al., 2021). There can though be concerns with the extent of patient benefits with CSII systems over multiple daily injections; however, again this is not universal (Mueller-Godeffroy et al., 2018; Blair et al., 2019). In view of the various published studies, the Endocrine Society guidelines published in 2018 recommended CSII systems use over multiple daily insulin injections in patients with T1DM who are not achieving their glycaemic targets, alternatively achieving their targets but continuing to experience severe hypoglycaemia, require increased flexibility with their insulin administration due to a variety of factors, or seek improved treatment satisfaction (Peters et al., 2018). However, this must be accompanied by comprehensive training on pump management (Grunberger et al., 2021), with the associated costs factored into any funding decision making. It is recognised though that these developments associated with higher costs are difficult to justify within healthcare systems struggling to fund even NPH and similar insulins, along with accompanying monitoring equipment, on a regular basis (Godman et al., 2020b).

Having said this, recent studies have suggested there are both clinical and economic benefits with HCL systems including the MiniMed^™^ 670G HCL system versus CSII systems among both adolescents and adults (Rodbard, 2017). Roze et al. (2021) in their study projected that there would be an additional 1.73 quality-adjusted life years (QALYs) associated with the MiniMed^™^ HCL system versus CSII systems at 14.09 *vs.* 12.36 QALYs respectively (Roze et al., 2021). This increase in QALYs was aided by a reduced fear of hypoglycaemia with the MiniMed^™^ HCL system, with improved blood glucose control enhancing user satisfaction (Roze et al., 2021). However, the authors calculated that the lifetime costs for the MiniMed^™^ HCL system would be GB£35,425 (US$48,200) higher than CSII systems, giving an incremental cost-effectiveness ratio of £20,421 per QALY gained (US$27,850) (Roze et al., 2021). A higher cost per QALY was seen in Sweden at SEK500,000 (US$57,260) (Jendle et al., 2021).

This increased cost is a concern as CSII systems are appreciably more expensive than multiple daily administration of insulin without adding in HCL systems and any associated patient education (Heinemann and DeVries, 2016). However, the improvement in patient outcomes seen with the newer devices resulted in the National Institute for Health and Care Excellence (NICE) in the United Kingdom stating that patients with T1DM, whose HbA1c levels are not being optimised, should be offered insulin pumps (Roze et al., 2021). However, there can still be concerns with their value despite improvements in treatment satisfaction (Muñoz-Velandia et al., 2019). Despite this recommendation though, there is a concern that the use of insulin pumps in practice in the United Kingdom is low compared to other Western European countries. This alongside concerns with funding CSII systems, as well as CSII and HCL systems, in CEE countries given existing issues with the routine funding of biological therapies in these countries (Baumgart et al., 2019; Roze et al., 2021). Having said this, there is increased funding for long-acting insulin analogues among CEE countries in recent years to improve patient care (Godman et al., 2021d).

It is imperative across all countries that patients with diabetes requiring insulin, especially children and adolescents, are well managed given the burden of the disease and its potential complications, as well as the additional burden during their education. Issues of funding and choices for developments such as CGM and CSII systems with or without HCLs need to be placed in context given current inconsistencies in funding decisions across disease areas and countries especially among higher income countries, and the increasing burden of diabetes as well as its complications worldwide. This recognises that such discussions are more difficult in some LMICs where patients are struggling to fund even standard insulins within public healthcare systems, with these discussions continuing exacerbated by the recent COVID-19 pandemic (Ogunleye et al., 2020). However, this is not universal as seen with growing use of long-acting insulin analogues, including biosimilars, in a number of Asian and CEE countries (Godman et al., 2021c; Godman et al., 2021d; Tubic et al., 2021).

### Considerations in Choices for Funding Treatments Including Insulins and Devices

We are aware that global expenditure on medicines has risen appreciably in recent years, and is estimated to reach US$1.5 trillion by 2023, representing an annual compounded growth rate of 3–6% (IQVIA, 2019). This growth rate is driven by many factors including increasing expenditure on new premium-priced medicines especially for cancer and orphan diseases as both are emotive disease areas (Haycox, 2016; Luzzatto et al., 2018; Morgan et al., 2020; Godman et al., 2021e; Godman et al., 2021g). The costs of new cancer medicines have risen two-fold in recent years, with the reimbursed price per life year gained rising four fold in the past 20 years after adjusting for inflation (Godman et al., 2021g). This is despite limited health gain with most new high-priced cancer medicines, with high prices being granted even with limited clinical information (Cohen, 2017; Pontes et al., 2020; Godman et al., 2021g). In high-income countries, we are also seeing new treatments for patients with orphan diseases being funded at ever increasing costs (Luzzatto et al., 2018). This includes patients with cystic fibrosis with a cost per QALY for lumacaftor–ivacaftor (Orkambi^®^) of Ca$3.6 million (Hollis, 2019). Previously, new medicines for enzyme replacement therapy and Pompe’s disease have been reimbursed up to Euro15 million per QALY (Simoens et al., 2013; Godman et al., 2018). More recently, there have been active discussions across countries including the United Kingdom regarding the funding of new treatments for infants with spinal muscular atrophy at a cost of GB£1.75million (US$2.38million) per dose (NHS, 2021). Prior to this, the United Kingdom Government established a separate fund covering the cost of new high priced cancer medicines that NICE found of limited value (Author Anonymous, 2010). This separate budget was taken out of existing funds prompting the Lancet to suggest such decisions were “intellectually bankrupt” within a universal healthcare system as funds are transferred from other disease areas without robust justification (Author Anonymous, 2010).

We do not see this in other disease areas where for instance in Scotland, expenditure on medicines for coronary vascular disease, rheumatoid arthritis and mental health, have fallen in recent years despite growing use through the availability of low cost multiple sourced medicines and biosimilars (Bennie et al., 2013; Leporowski et al., 2018; Godman et al., 2019; Godman et al., 2021e).

Such activities and growth rates in prices and expenditures for new medicines for oncology and orphan diseases are difficult to sustain under opportunity cost considerations within universal healthcare systems (Barrett et al., 2006; Godman et al., 2021g), necessitating a closer look at the most effective use of current resources. This is imperative with publications advocating greater spending on new medicines for cancer and orphan diseases in CEE countries despite limited health gain for most new cancer medicines (Cohen, 2017; Tomić et al., 2018; Cufer et al., 2020; Malinowski et al., 2020). The situation is different among LMICs, including sub-Saharan Africa, where in the case of patients with diabetes, key initial considerations include early diagnosis as well as availability and access to standard insulins (Godman et al., 2021b).

More consistent decision making in higher income countries, as well as potentially a number of CEE countries, this could involve reviewing increased funding for new cost-effective treatments and devices for patients in other disease areas apart from oncology and orphan diseases. This could incorporate patients with diabetes to improve their care and subsequent quality-of-life. As a result, improve consistency in decision making. This could include CGM and CSII systems, including potential HCL additions, where there are concerns with their funding and use in recommended situations in higher-income countries.

## Conclusion and Recommendations

The current COVID-19 pandemic appears to be increasing prevalence rates for non-communicable diseases (NCDs) and their associated costs (Kluge et al., 2020). While there is always uncertainty regarding long-term projections on outcomes and expenditure based on short-term data, especially if new medicines are launched with limited data, we need to consult more with patients, the public and other key stakeholders to debate, and prioritise funding decisions within finite resources. This should help avoid inconsistent and emotive decision making and maximise health outcomes for patients within available resources. This is particularly important in patients with diabetes given the growing burden in terms of both morbidity and mortality as well as costs (Fifer et al., 2018; Muñoz-Velandia et al., 2019; González-González et al., 2021), and should be part of general moves towards a quality health service as part of universal healthcare (WHO, 2018). Prioritising investment in one disease area where there are concerns with costs and value will necessarily have a detrimental impact on other disease areas.

This goes hand-in-hand with encouraging transferable learning between countries, advocacy and compliance with key areas, as well as greater evidence-based approaches to decision making (Godman et al., 2021e). This is imperative following the considerable unintended consequences associated with COVID-19 including a rise in NCDs and an associated increase in morbidity and mortality. Furthermore, ongoing debates especially across Europe regarding pricing and funding approaches, as well as greater pricing transparency for new cancer medicines and those for orphan diseases (Godman et al., 2018; Kluge et al., 2020; Godman et al., 2021e; Godman et al., 2021g). This is essential given ongoing debates about the future sustainability of healthcare systems (Godman et al., 2021g). We will continue to monitor these situations, and report on them, to stimulate debates in important disease areas including diabetes where there are inconsistencies in decision making. This especially with all countries being encouraged to instigate universal healthcare by 2030 embraced by all European countries with necessary transparency and consistency in decision making (WHO, 2018; Cerf, 2019).

## Data Availability

Publicly available datasets were analyzed in this study. Additional information can be found in the pertinent references here.
